# Clonal Relatedness and Transferable Resistance in Carbapenem-Resistant *Klebsiella pneumoniae*: An Integrated Molecular Epidemiological Study

**DOI:** 10.3390/microorganisms14102181

**Published:** 2026-09-29

**Authors:** Cihat Öztürk, Özlem Ünaldi, Zekiye Bakkaloğlu, Fatma Filiz Coşkun-Ari, Elif Sevim

**Affiliations:** 1 Department of Medical Microbiology, Faculty of Medicine, Kırşehir Ahi Evran University, Kırşehir 40100, Türkiye; 2 Department of Microbiology Reference Laboratory and Biological Products, Ministry of Health General Directorate of Public Health, Ankara 06100, Türkiye; ozlemunaldii@gmail.com (Ö.Ü.); zekiye.yb@gmail.com (Z.B.); 3 Department of Medical Biology, Faculty of Medicine, Kirsehir Ahi Evran University, Kirşehir 40100, Türkiye; filiz.ari@ahievran.edu.tr (F.F.C.-A.); esevim@ahievran.edu.tr (E.S.)

**Keywords:** *Klebsiella pneumoniae*, PFGE, *qnrD*, Conjugative plasmid transfer

## Abstract

Background: Carbapenem-resistant *Klebsiella pneumoniae* (CRKP) represents a major global clinical threat driven by both clonal relatedness and horizontal transfer of antimicrobial resistance determinants. This study aimed to characterize CRKP isolates through an integrated analysis of phenotypic resistance, resistance genes, conjugative transferability, plasmid replicon backgrounds, and clonal relatedness. Methods: A total of 62 confirmed CRKP clinical isolates were investigated. Antimicrobial susceptibility profiles were evaluated according to EUCAST criteria. Carbapenemase, β-lactamase, extended-spectrum β-lactamase-associated, and plasmid-mediated quinolone resistance (PMQR) determinants were screened by PCR. Conjugation experiments were performed using *Escherichia coli* J53 as the recipient strain. Plasmid replicon typing was conducted on transconjugants by PCR-based replicon typing, and clonal relatedness was assessed by pulsed-field gel electrophoresis (PFGE). Results: All isolates were resistant to ciprofloxacin, levofloxacin, imipenem, ertapenem, meropenem and ampicillin. The most prevalent resistance determinants were *oqxA* and *oqxB* (100%), *blaSHV* (96.8%), *blaCTX-M-1* (93.5%), *blaTEM* and *aac(6′)-Ib-cr* (85.5% each), *qnrD* (69.4%), and *blaOXA-48* (66.1%). Conjugative transfer was detected in 14 of the 62 isolates (22.6%) under the experimental conditions used. *blaOXA-48*, *blaCTX-M-1*, *blaTEM*, and *blaSHV* were detected in all transconjugants, whereas *aac(6′)-Ib-cr* was transferred in only one. IncFIB was the predominant replicon among transconjugants (13/14, 92.9%). PFGE analysis of 57 isolates identified 24 groups and 41 pulsotypes, with one predominant group comprising 29/57 isolates; conjugation-positive isolates were distributed across multiple PFGE backgrounds. Conclusions: The CRKP population showed extensive multidrug resistance together with both clonal clustering and horizontally transferable β-lactam resistance. The unusually high prevalence of sequence-confirmed *qnrD* represents a distinctive finding and, to the best of our knowledge, the first report of *qnrD* in clinical *Klebsiella pneumoniae* isolates from Türkiye. The predominance of IncFIB among transconjugants and the distribution of conjugation-positive isolates across different PFGE backgrounds support the coexistence of clonal dissemination and horizontal resistance transfer in the local CRKP population.

## 1. Introduction

*Klebsiella pneumoniae* (*K. pneumoniae*) is a Gram-negative, encapsulated opportunistic pathogen responsible for a broad spectrum of healthcare- and community-associated infections, including pneumonia, urinary tract infections, bloodstream infections, and liver abscesses [1]. Over the past two decades, the emergence and global dissemination of multidrug-resistant and carbapenem-resistant lineages have transformed *K. pneumoniae* into one of the most urgent threats in clinical microbiology. In the 2024 World Health Organisation (WHO)’s Bacterial Priority Pathogens List, carbapenem-resistant *K. pneumoniae* was classified within the critical-priority tier and ranked first among the 24 antibiotic-resistant bacterial pathogens evaluated, with a total prioritization score of 84% [2]. This ranking reflects its substantial clinical burden, transmissibility, increasing resistance trends, and the limited therapeutic options available for infections caused by carbapenem-resistant isolates [2].

Carbapenem resistant in *K. pneumoniae* (CRKP) is predominantly mediated by acquired carbapenemases belonging to Ambler class A, including KPC and certain GES variants; class B metallo-β-lactamases, including NDM, VIM, and IMP; and class D OXA-48-like enzymes. These mechanisms may be accompanied by reduced outer-membrane permeability and increased efflux activity, particularly in isolates producing extended-spectrum β-lactamases or other β-lactamases. OXA-48-like carbapenemases have particular epidemiological relevance in Türkiye, where the prototype OXA-48 enzyme was first identified in a clinical *K. pneumoniae* isolate [3]. OXA-48-like carbapenemases have historically been the predominant carbapenemase type reported in Türkiye and remain highly prevalent in recent CRKP studies, although their frequency varies according to center, region, and study period [4,5,6].

Carbapenemase-producing *K. pneumoniae* isolates frequently carry extended-spectrum β-lactamase genes, including *blaSHV*, *blaTEM*, and *blaCTX-M*, together with plasmid-mediated quinolone resistance determinants such as *qnr* genes and *aac(6′)-Ib-cr*. The *oqxAB* efflux system may additionally contribute to reduced quinolone susceptibility, although *oqxAB* may be chromosomally encoded or plasmid-associated in *K. pneumoniae* [7]. Because many antimicrobial resistance determinants are embedded within plasmids, transposons, integrons, and other mobile genetic elements, their detection alone does not establish their capacity for horizontal dissemination. Conjugation experiments provide functional evidence of transferability under defined laboratory conditions, whereas plasmid replicon typing enables the classification and epidemiological tracking of resistance-associated plasmid backgrounds. Both conjugative and mobilizable plasmids are recognized as key vehicles for the horizontal transfer of antimicrobial resistance determinants among Enterobacteriaceae [8,9]. In *K. pneumoniae* specifically, mobilizable plasmids lacking a complete conjugation machinery have been shown to co-transfer with helper conjugative plasmids, contributing substantially to the dissemination of both resistance and virulence determinants [10].

Clonal analysis is essential for assessing whether carbapenem resistance is associated with genetically related or diverse strain backgrounds. PFGE provides high discriminatory power for investigating local epidemiological relationships and was therefore selected as the primary typing method in the present study [11]. Previous molecular epidemiological studies from Türkiye have demonstrated both the predominance of particular high-risk lineages in some settings and substantial clonal heterogeneity in others [12,13,14], highlighting the importance of local clonal surveillance of CRKP. Nevertheless, studies integrating phenotypic susceptibility, resistance-gene profiling, conjugative transfer, plasmid replicon typing, and PFGE within the same isolate collection remain limited.

In this study, we aimed to characterize carbapenem-resistant *K. pneumoniae* isolates using resistance-gene profiling, conjugation experiments, plasmid replicon typing, and PFGE-based clonal analysis, and to evaluate clonal relatedness together with the presence of horizontally transferable resistance determinants.

## 2. Materials

### 2.1. Bacterial Isolates

Clinical *K. pneumoniae* isolates recovered between September 2021 and January 2022 at Kırşehir Ahi Evran University Training and Research Hospital were retrospectively evaluated. The isolates originated from patients admitted to intensive care units and various clinical departments, including internal medicine, infectious diseases, pulmonology, neurosurgery, cardiology, nephrology, urology, and emergency medicine. The final collection comprised isolates recovered from urine (*n* = 38), blood cultures (*n* = 17), wound cultures (*n* = 3), sputum (*n* = 3), and catheter culture (*n* = 1).

Antimicrobial susceptibility testing of the original clinical isolates, including determination of the minimum inhibitory concentrations (MICs) of ertapenem, imipenem, and meropenem, was performed using the BD Phoenix automated identification and antimicrobial susceptibility testing system. The original MIC values were retrieved from the hospital’s KARMED laboratory information system and retrospectively interpreted according to the EUCAST clinical breakpoint tables, version 13.0 [15]. 

During the study period, records of 256 *K. pneumoniae* isolates were retrospectively reviewed. Seventy-two non-duplicate isolates classified as resistant to at least one carbapenem (ertapenem, imipenem, or meropenem) on the basis of their original MIC results were identified and retrieved from the laboratory stock culture collection. Following transport under cold-chain and aseptic conditions, the stored isolates were subcultured onto LB agar supplemented with meropenem (10 µg/mL) and incubated at 37 °C for 18–24 h. This procedure was used exclusively as a selective recovery step following long-term storage and was not intended or interpreted as a standardized confirmatory antimicrobial susceptibility test. Of the 72 candidate isolates, 62 yielded viable growth under these selective conditions and were included in the molecular analyses. The remaining 10 isolates were excluded because they could not be recovered under the conditions used; their failure to grow was not interpreted as evidence of carbapenem susceptibility. The carbapenem-resistant classification of the 62 included isolates was based exclusively on their original MIC-based susceptibility results, as detailed in Supplementary Table S4. Only one isolate per patient was included, resulting in a final study population of 62 non-duplicate CRKP isolates from 62 individual patients.

The sodium azide resistant *Escherichia coli* (*E. coli*) J53 (*met pro Azi*^r^) reference strain used as the recipient cell in conjugation was obtained from the culture collection of the Kırşehir Ahi Evran University, Faculty of Medicine, Department of Medical Biology (Prof. Dr. Elif Sevim). The resistance profile of the *E. coli* J53 strain against the antibiotics used was determined using the disk diffusion method.

### 2.2. DNA Extraction

Bacterial stocks were transported under aseptic conditions from the Kırşehir Ahi Evran University Training and Research Hospital Microbiology Laboratory culture collection to the Kırşehir Ahi Evran University Faculty of Medicine, Department of Medical Microbiology Laboratory, where bacterial revival, DNA extraction, and PCR-based resistance analyses were performed. Frozen glycerol stocks and *E. coli* J53 were sub-cultured onto LB agar and incubated aerobically at 37 °C for 18–24 h to obtain pure, viable cultures prior to any downstream analysis.

Template DNA was extracted from freshly revived colonies using the boiling method, as previously described by Çam et al. [16]. Briefly, a full loopful of fresh culture was suspended in 300 µL of sterile distilled water in a microcentrifuge tube and vortexed to obtain a homogeneous suspension. The suspension was boiled in a water bath at 100 °C for 10 min, followed by centrifugation at 14,800 rpm for 10 min. The resulting supernatant was collected and used as template DNA for all PCR-based analyses. The integrity and suitability of the extracted DNA for downstream PCR were assessed by agarose gel electrophoresis, together with successful amplification of expected target products using validated positive controls. Extracted DNA was stored at −20 °C until use.

### 2.3. PCR Analysis

All isolates and *E. coli* J53 were screened using the PCR method for the presence of carbapenemase-encoding genes (*blaKPC*, *blaNDM*, *blaIMP*, *blaVIM*, *blaOXA-48*, *blaOXA-23*, *blaOXA-51*, *blaOXA-58*, *blaOXA-40*), β-lactamase and ESBL associated genes (*blaSHV*, *blaTEM*, *blaCTX-M-1*, *blaCTX-M-2*, *blaGES*, *blaPER-1*, *blaVEB*, *blaOXA-1*), and plasmid-mediated quinolone resistance (PMQR) determinants (*qnrA*, *qnrB*, *qnrC*, *qnrD*, *qnrS*, *qepA*, *oqxA*, *oqxB*, *aac(6′)-Ib-cr*), using gene-specific primers as given in Supplementary Table S1 [8,17,18,19,20,21,22,23,24,25,26,27,28,29,30,31,32,33].

Singleplex PCR reactions were performed for detection of carbapenemase genes (*blaKPC*, *blaNDM*, *blaIMP*, *blaVIM*, *blaOXA-48*, *blaOXA-23*, *blaOXA-51*, *blaOXA-58*, *blaOXA-40*), and β-lactamase and ESBL associated genes (*blaSHV*, *blaTEM*, *blaCTX-M-1*, *blaCTX-M-2*, *blaGES*, *blaPER-1*, *blaVEB*, *blaOXA-1*) in PCR tubes with a final volume of 25 µL, containing 12.5 µL 2X Master Mix (HY-K0531, MedChemExpress, Monmouth Junction, NJ, USA), 0.5 µL forward (0.2 µM) and reverse (0.2 µM) primers, 5 µL template DNA and 6.5 µL nuclease-free water. For all target genes, amplification began with an initial denaturation step at 95 °C for 3 min and consisted of 35 cycles, with a final extension at 72 °C. As shown in Supplementary Table S2, temperature and time of the denaturation, annealing, extension and final extension steps varied depending of the target genes. PCR products were analyzed on 1% agarose gels containing ethidium bromide by one hour- electrophoresis performed at 110 Volt. Amplicons were then visualized under ultraviolet illumination and their sizes were compared with a molecular weight marker (HY-K0803, MedChemExpress, Monmouth Junction, NJ, USA) to confirm expected product size for each target gene.

PMQR determinants were screened using multiplex and singleplex PCR assays according to the protocols provided in Supplementary Table S2 [34,35]. A four-target multiplex assay was used for *qnrA*, *qnrB*, *qnrS*, and *aac(6′)-Ib-cr*. Each 25 µL reaction contained 13 µL of PCR master mix (HY-K0531, MedChemExpress, Monmouth Junction, NJ, USA), 5 µL of template DNA, 0.1 µL (0.04 µM) each of the forward and reverse primers for all four targets, and 6.2 µL of nuclease-free water. A separate three-target multiplex assay was used for *oqxA*, *oqxB*, and *qnrD*, containing 13 µL of PCR master mix 5 µL of template DNA, 0.2 µL (0.08 µM) each of the *oqxA* and *qnrD* forward and reverse primers, 0.1 µL (0.04 µM) each of the *oqxB* forward and reverse primers, and 6 µL of nuclease-free water. The *qepA* and *qnrC* genes were screened individually by singleplex PCR.

A validated positive control was included (except for *qnrC*) in PCR run for all target genes. Representative isolates yielding a PCR product of the expected size for each gene were selected and subjected to Sanger sequencing to confirm gene identity. 

### 2.4. Detection of Integron Gene-Cassette Regions

Class 1 and class 2 integron-associated gene-cassette regions were investigated by conventional PCR. For class 1 integrons, the variable region between the 5′- and 3′-conserved segments was amplified using the 5′-CS and 3′-CS primers described by Lévesque et al. [32]. For class 2 integrons, the gene-cassette region was amplified using the hep51 and hep74 primers described by White et al. [33]. These assays targeted the variable gene-cassette regions rather than the *intI1* or *intI2* integrase genes. Because cassette number and composition may vary among isolates, the expected amplicon size was variable rather than fixed. As integron screening was not the primary objective of this study, a dedicated reference positive control strain was not procured from an external source. Instead, an isolate previously confirmed to harbor both class 1 and class 2 integrons, obtained from the culture collection of the Department of Medical Microbiology, Kırşehir Ahi Evran University [35], was used as a positive control to verify the adequacy of the PCR reaction, and PCR-grade water was included as a no-template negative control in each run. PCR products were evaluated by agarose gel electrophoresis. Integron-associated PCR products were not subjected to Sanger sequencing.

### 2.5. Conjugation Experiments

Conjugation experiments were performed to assess the transferability of antimicrobial resistance determinants from CRKP donor isolates to sodium azide-resistant *E. coli* J53 (met pro Aziʳ) as the recipient strain, according to the method described by Ozgumus et al. [36].

In the conjugation experiments, CRKP isolates were used as donor cells, while *E. coli* J53 was used as the recipient strain. Donor and recipient cells were inoculated into 3 mL of antibiotic-free LB broth and incubated in a shaking incubator at 37 °C for 18–24 h under atmospheric conditions. Equal volumes (1:1) of the donor and recipient cultures were then mixed and incubated without shaking at 35 °C for 18–20 h.

For the selection of transconjugants, 100 µL of a 10^−1^ dilution of the conjugation mixture was inoculated onto the surface of EMB agar supplemented with sodium azide (100 µg/mL), to which the recipient *E. coli* J53 strain was resistant, and ampicillin (100 µg/mL), to which the donor cells were resistant. The inoculum was evenly spread over the agar surface using a sterile glass spreader and incubated at 35 °C for 18–24 h. Colonies exhibiting a metallic blue-green sheen were considered phenotypically presumptive transconjugants.

Presumptive transconjugant colonies were subsequently replica-plated onto four selective media with different compositions. Agar 1 was minimal agar containing only one of the amino acids, methionine or proline, for which the recipient *E. coli* J53 strain is auxotrophic. Agar 2 was EMB agar supplemented with sodium azide (100 µg/mL), to which the recipient strain was resistant. Agar 3 was EMB agar supplemented with ampicillin (100 µg/mL), to which the donor cells were resistant. Agar 4 was EMB agar supplemented with both ampicillin (100 µg/mL) and sodium azide (100 µg/mL). Phenotypically, colonies exhibiting a metallic green sheen that failed to grow on Agar 1 but grew on Agars 2, 3, and 4 were considered transconjugants.

Transconjugants were then characterized phenotypically by antimicrobial susceptibility testing and genotypically by gene-specific PCR. Phenotypic antimicrobial resistance profiles were determined using the Kirby–Bauer disk diffusion method. For genotypic characterization, transconjugant colonies were subcultured in LB broth containing sodium azide and ampicillin, and plasmid DNA was extracted using the GeneJET Plasmid Miniprep Kit (Thermo Fisher Scientific, Waltham, MA, USA) according to the manufacturer’s instructions. The plasmid DNA was used as the template for PCR screening of ESBL, carbapenemase, and quinolone-resistance genes in the transconjugants using the primer sets described in Supplementary Table S1. Whole-cell DNA was used separately for resistance-gene screening in the original *K. pneumoniae* donor isolates.

### 2.6. PCR-Based Plasmid Replicon Typing

Plasmid incompatibility (Inc) groups associated with conjugative transfer were determined by PCR-based replicon typing (PBRT), following the protocol of Carattoli et al. [8]. Eighteen replicon-specific primer pairs, organized into five multiplex and three simplex PCR reactions, were used to screen for the FIA, FIB, FIC, HI1, HI2, I1-Iγ, L/M, N, P, W, T, A/C, K, B/O, X, Y, F, and FIIA/FIIS incompatibility groups (Supplementary Table S1). Replicon typing was performed on template DNA obtained from confirmed transconjugants to identify plasmid replicon backgrounds associated with the transferred resistance phenotype. PBRT was used to identify plasmid replicon types present in the transconjugants; however, this method does not establish the physical localization of individual resistance genes on a specific plasmid replicon.

### 2.7. Pulsed-Field Gel Electrophoresis (PFGE)

Clonal relatedness among the *K. pneumoniae* isolates was investigated by pulsed-field gel electrophoresis (PFGE) at the National Antimicrobial Resistance Surveillance Laboratory, Department of Microbiology Reference Laboratories and Biological Products, General Directorate of Public Health, Türkiye. PFGE was performed according to a previously described protocol with two minor modifications [37]. First, the incubation period in the lysis buffer was extended overnight. Second, thiourea was added to the electrophoresis running buffer to improve PFGE profile quality and reduce DNA degradation during electrophoresis. Specifically, 1 mL of a 1 M thiourea stock solution was added to 2 L of running buffer, resulting in a final thiourea concentration of 0.5 mM.

Bacterial suspensions were prepared from overnight cultures grown on sheep blood agar, and the cell density was adjusted to an optical density of approximately 1.0 at 600 nm (OD600). The bacterial suspensions were mixed with an equal volume of 1% agarose containing proteinase K to prepare agarose plugs. Bacterial cells embedded within the plugs were lysed using a lysis buffer containing Tris-EDTA, sarkosyl, and proteinase K, with incubation performed overnight as described above. Following lysis, the plugs were washed sequentially with ultrapure water and Tris-EDTA buffer.

After washing, chromosomal DNA embedded within the plugs was digested with 30 U of *XbaI* restriction endonuclease (Fermentas Inc. [Thermo Fisher Scientific], Glen Burnie, MD, USA). The digested plugs were subsequently loaded onto a 1% pulsed-field-grade agarose gel prepared in 0.5× TBE buffer (Tris base, boric acid, and EDTA). Electrophoresis was performed using a CHEF-DR III system (Bio-Rad Laboratories, Inc., Hercules, CA, USA) under the following conditions: initial switch time, 5 s; final switch time, 20 s; included angle, 120°; electric field strength, 6 V/cm; temperature, 15 °C; and run time, 19 h. Thiourea was included in the running buffer at a final concentration of 0.5 mM.

Following electrophoresis, the gels were stained with 0.5 µg/mL ethidium bromide, and DNA banding patterns were visualized using a ChemiDoc MP Imaging System (Bio-Rad Laboratories, Inc., Hercules, CA, USA). *Salmonella enterica* serovar Braenderup H9812 (ATCC BAA-664) was used as the molecular size reference standard for normalization and inter-gel comparison of PFGE profiles.

PFGE banding patterns were analyzed using BioNumerics software version 7.5 (Applied Maths, Sint-Martens-Latem, Belgium). Similarity between isolates was calculated using the Dice similarity coefficient, and dendrograms were generated using the unweighted pair group method with arithmetic mean (UPGMA). Band-position tolerance and optimization values were set at 1.5% and 1.0%, respectively. A Dice similarity threshold of ≥85% was used to define PFGE groups; isolates showing ≥85% similarity were therefore assigned to the same PFGE group, whereas isolates displaying indistinguishable banding patterns were classified as the same pulsotype. Epidemiological relatedness was further interpreted according to the criteria proposed by Tenover et al. [11], based on differences in PFGE banding patterns. Isolates with no band differences were considered indistinguishable, those differing by 2–3 bands were considered closely related, those differing by 4–6 bands were considered possibly related, and those differing by ≥7 bands were considered different. These criteria were used to operationalize the interpretation of PFGE profiles and to assess the epidemiological relatedness of the isolates.

### 2.8. Statistical Analysis

The study was designed as a descriptive molecular epidemiological investigation; therefore, no patient-level demographic, clinical, treatment, or outcome variables were included in the analysis, and no inferential statistical comparisons involving patient-related factors were performed. Antimicrobial resistance frequencies, resistance-gene distributions, conjugation results, plasmid replicon types, and PFGE findings were summarized using counts and percentages. For important proportions, including the prevalence of resistance determinants, 95% confidence intervals (CIs) were calculated using the Wilson score method [38]. These confidence intervals were used as descriptive measures of precision for the observed proportions within the study isolate collection and were not intended for patient-level clinical inference.

## 3. Results

### 3.1. Antimicrobial Susceptibility Profiles

Antimicrobial susceptibility results were available for all 62 CRKP isolates. All isolates were resistant to ciprofloxacin, levofloxacin, imipenem, ampicillin, ertapenem, and meropenem (62/62, 100% for each antimicrobial). High resistance rates were also observed for amoxicillin/clavulanic acid and cefuroxime (52/62, 83.9% each), piperacillin/tazobactam (50/62, 80.6%), cefoxitin (49/62, 79.0%), ceftriaxone (48/62, 77.4%), and ceftazidime (47/62, 75.8%). Resistance to trimethoprim/sulfamethoxazole, gentamicin, and amikacin was detected in 44/62 (71.0%), 43/62 (69.4%), and 42/62 (67.7%) isolates, respectively (Supplementary Table S3).

### 3.2. Distribution of Antimicrobial Resistance Genes

**Figure 1:**
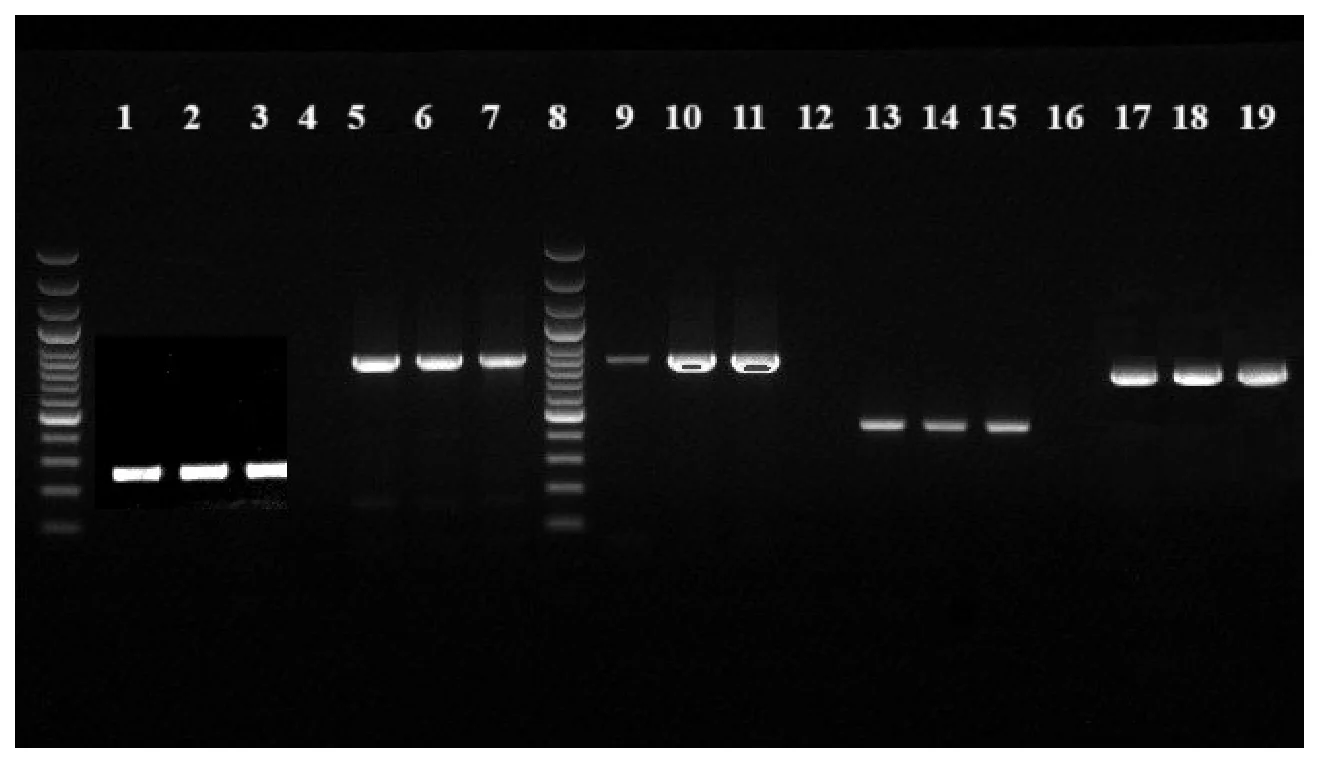
Representative agarose gel electrophoresis of PCR products for β-lactamase and carbapenemase genes. M and lane 8, DNA molecular size markers; lane 1, positive control for *blaCTX-M-1*; lanes 2–3, *blaCTX-M-1*-positive isolates (260 bp); lane 4, empty; lane 5, positive control for *blaTEM*; lanes 6–7, *blaTEM*-positive isolates (847 bp); lane 9, positive control for *blaSHV*; lanes 10–11, *blaSHV*-positive isolates (843 bp); lane 12, empty; lane 13, positive control for *blaOXA-1*; lanes 14–15, *blaOXA-1*-positive isolates (427 bp); lane 16, empty; lane 17, positive control for *blaOXA-48*; and lanes 18–19, *blaOXA-48*-positive isolates (743 bp).

**Figure 2:**
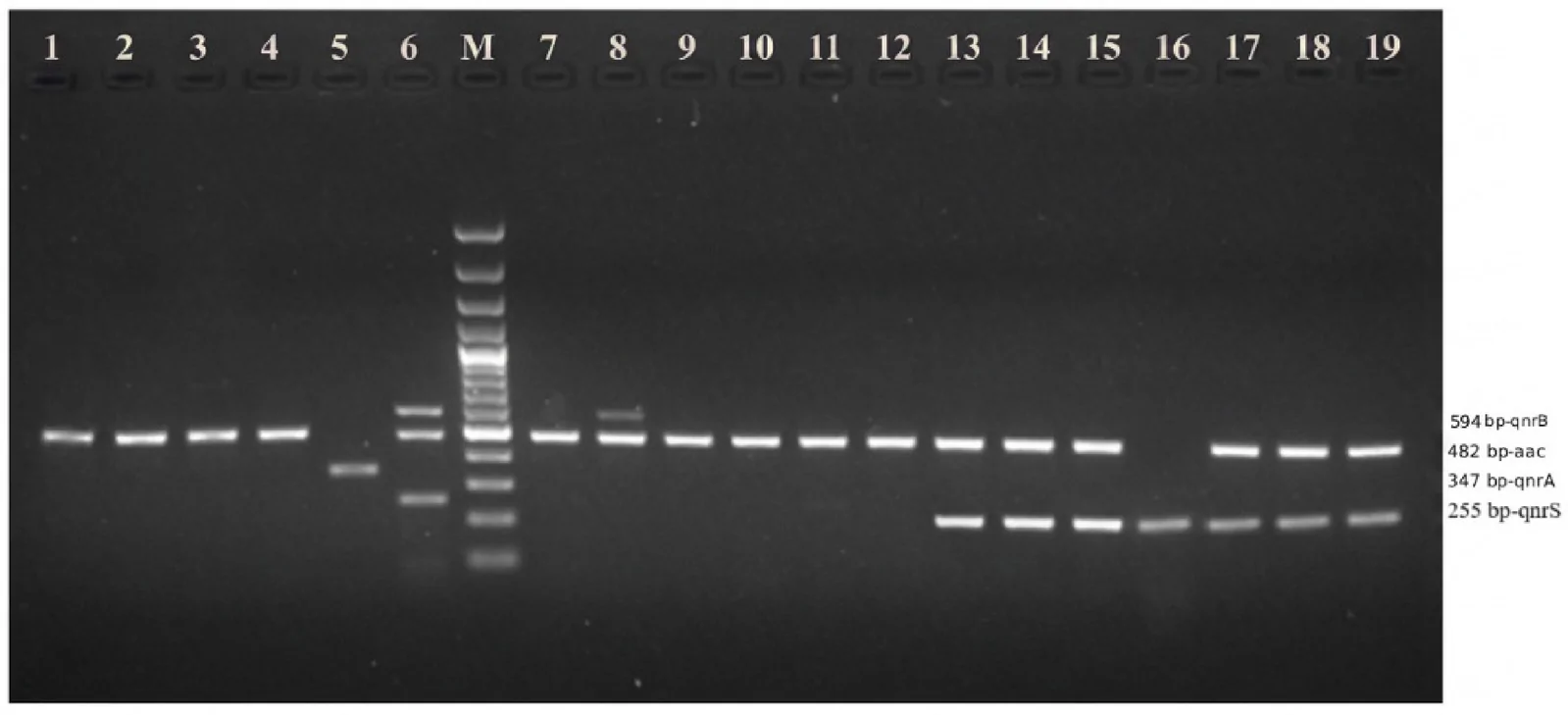
Representative agarose gel electrophoresis of multiplex PCR products for plasmid-mediated quinolone resistance genes. M, DNA molecular size marker. Amplification products correspond to *qnrB* (594 bp), *aac(6′)-Ib-cr* (482 bp), *qnrA* (347 bp), and *qnrS* (255 bp). Lane 5 represents the *qnrA* positive control, and lane 6 represents the combined *qnrB*, *aac(6′)-Ib-cr*, and *qnrS* positive control. The remaining lanes represent the tested isolates exhibiting different combinations of these resistance genes.

**Figure 3:**
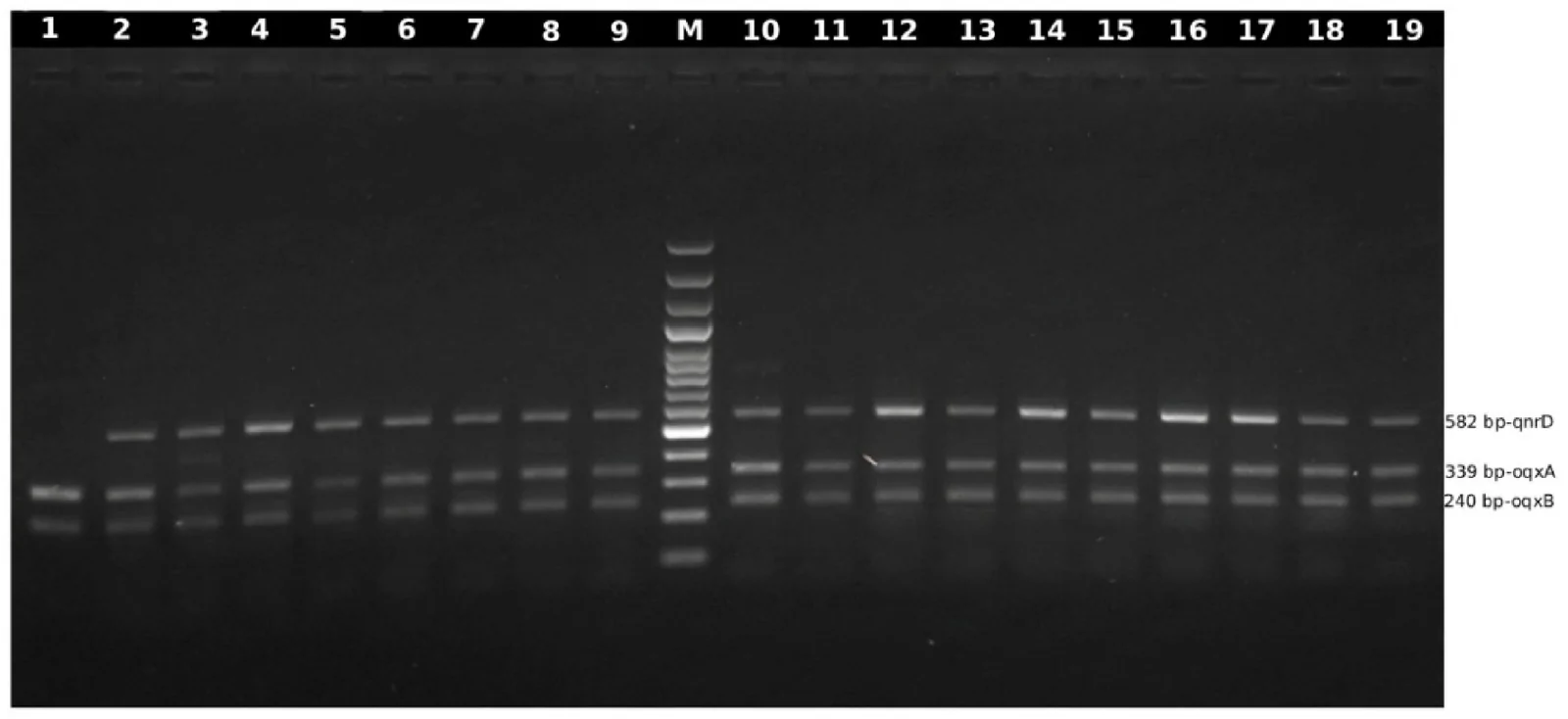
Representative agarose gel electrophoresis of multiplex PCR (mPCR) products for plasmid-mediated quinolone resistance genes. M, DNA molecular size marker. The upper, middle, and lower bands correspond to *qnrD* (582 bp), *oqxA* (339 bp), and *oqxB* (240 bp), respectively. Lane 10 represents the positive control, while the remaining lanes represent the tested isolates showing simultaneous amplification of these genes.

Among the 62 CRKP isolates, *oqxA* and *oqxB* were detected in all isolates (62/62, 100%). Among the β-lactamase genes, *blaSHV* was detected in 60/62 (96.8%) isolates, followed by *blaCTX-M-1* in 58/62 (93.5%), *blaTEM* in 53/62 (85.5%), and *blaOXA-1* in 29/62 (46.8%). The carbapenemase gene *blaOXA-48* was identified in 41/62 (66.1%) isolates, whereas *blaNDM* was detected in 3/62 (4.8%). Among the PMQR determinants, *aac(6′)-Ib-cr* was detected in 53/62 (85.5%) isolates, *qnrD* in 43/62 (69.4%), *qnrS* in 24/62 (38.7%), and *qnrB* in 3/62 (4.8%) (Table 1, Figure 1, Figure 2 and Figure 3). All *blaOXA-1*-positive isolates also harbored *blaSHV*, *blaCTX-M-1*, *aac(6′)-Ib-cr*, *oqxA*, and *oqxB*, while *blaTEM* and *blaOXA-48* indicating frequent co-occurrence of these resistance determinants within the same isolates. No positive results were obtained for *blaCTX-M-2*, *blaGES*, *blaKPC*, *blaIMP*, *blaVIM*, *blaVEB*, *blaPER-1*, *blaOXA-23*, *blaOXA-58*, *blaOXA-51*, *blaOXA-40*, *qnrA*, *qnrC*, or *qepA* (Supplementary Table S3). Given its high prevalence, one representative *qnrD*-positive *K. pneumoniae* isolate was confirmed by bidirectional Sanger sequencing. BLASTn analysis of the consensus sequence demonstrated 99.63% nucleotide identity and 97% query coverage with the closest matching GenBank reference sequence (accession no. CP071778), confirming the identity of the amplified product as *qnrD*. The sequence generated in the present study has not yet been assigned a GenBank accession number. 

### 3.3. Integron-Associated Gene-Cassette Regions

PCR amplification of the class 1 integron-associated gene-cassette region was observed in two isolates (Kp21, Kp36), whereas no amplification of the class 2 integron cassette region was detected. Kp36 belonged to PFGE group 1 (pulsotype 2). PFGE data were not available for Kp21.

### 3.4. Conjugation Experiments

The antimicrobial susceptibility testing results showed that the *E. coli* J53 strain was susceptible to all antibiotics tested and did not harbor any of the resistance genes screened by PCR.

Conjugative transfer was detected in 14 of the 62 CRKP isolates (22.6%) under the experimental conditions used. No transconjugants were recovered from the remaining 48 donor isolates under these conditions. The conjugation-positive donor isolates were Kp35, Kp42, Kp43, Kp45, Kp47, Kp50, Kp51, Kp55, Kp56, Kp58, Kp59, Kp60, Kp61 and Kp62; their corresponding transconjugants are hereafter referred to as TcKp35–TcKp62, respectively (Table 2).

All 14 transconjugants were resistant to ampicillin, amoxicillin/clavulanic acid, cefuroxime, ertapenem, meropenem, imipenem, and piperacillin/tazobactam [14/14 (100%) for each antimicrobial]. Ceftriaxone resistance was detected in 9/14 (64.3%) transconjugants, and ceftazidime resistance was similarly detected in 9/14 (64.3%) transconjugants, whereas gentamicin resistance was detected in only 1/14 (7.1%). None of the transconjugants retained the ciprofloxacin, levofloxacin, or amikacin resistance phenotypes observed in the corresponding donor isolates. Despite its high prevalence among the clinical isolates, *qnrD* was not transferred under the conjugation conditions used.

Genotypic analysis of the transconjugants showed that *blaTEM*, *blaSHV*, *blaCTX-M-1*, and *blaOXA-48* were detected in all 14 transconjugants [14/14 (100%)]. The *aac(6′)-Ib-cr* determinant was detected only in TcKp42 [1/14 (7.1%)].

### 3.5. Plasmid Replicon Typing

Plasmid replicon typing of the transconjugants obtained from the 14 conjugation-positive donor isolates showed that IncFIB was the predominant replicon, being detected in 13/14 (92.9%) transconjugants. FrepB was detected together with IncFIB in TcKp42 and TcKp45, whereas IncY was detected in TcKp35. No other replicon types among the screened targets were detected (Table 2).

### 3.6. Clonal Relatedness

PFGE analysis yielded interpretable profiles for 57 of the 62 isolates. PFGE data were unavailable for Kp20, Kp21, Kp51, Kp55, and Kp60; these isolates were therefore excluded from PFGE-based clustering analyses. Despite repeated attempts, interpretable PFGE banding patterns could not be obtained for these five isolates because of inadequate restriction profiles.

Based on the dendrogram, the isolates were distributed into 24 PFGE groups and 41 pulsotypes. PFGE group 1 was the predominant group and included 29/57 isolates (50.9%), whereas the remaining isolates were distributed among PFGE groups 2–24.

Within PFGE group 1, several pulsotypes were shared by multiple isolates. Pulsotype 2 was the most frequent and included isolates 28, 36, 18, 19, 3, 4, 6, and 45. Pulsotype 13 included isolates 31, 14, 15, and 50, while pulsotype 1 included isolates 32, 35, and 44. Pulsotypes 6 and 7 each comprised two isolates. Outside PFGE group 1, pulsotype 35 included isolates 41 and 46, and pulsotype 36 included isolates 58 and 59. The remaining pulsotypes were represented by single isolates.

**Figure 4:**
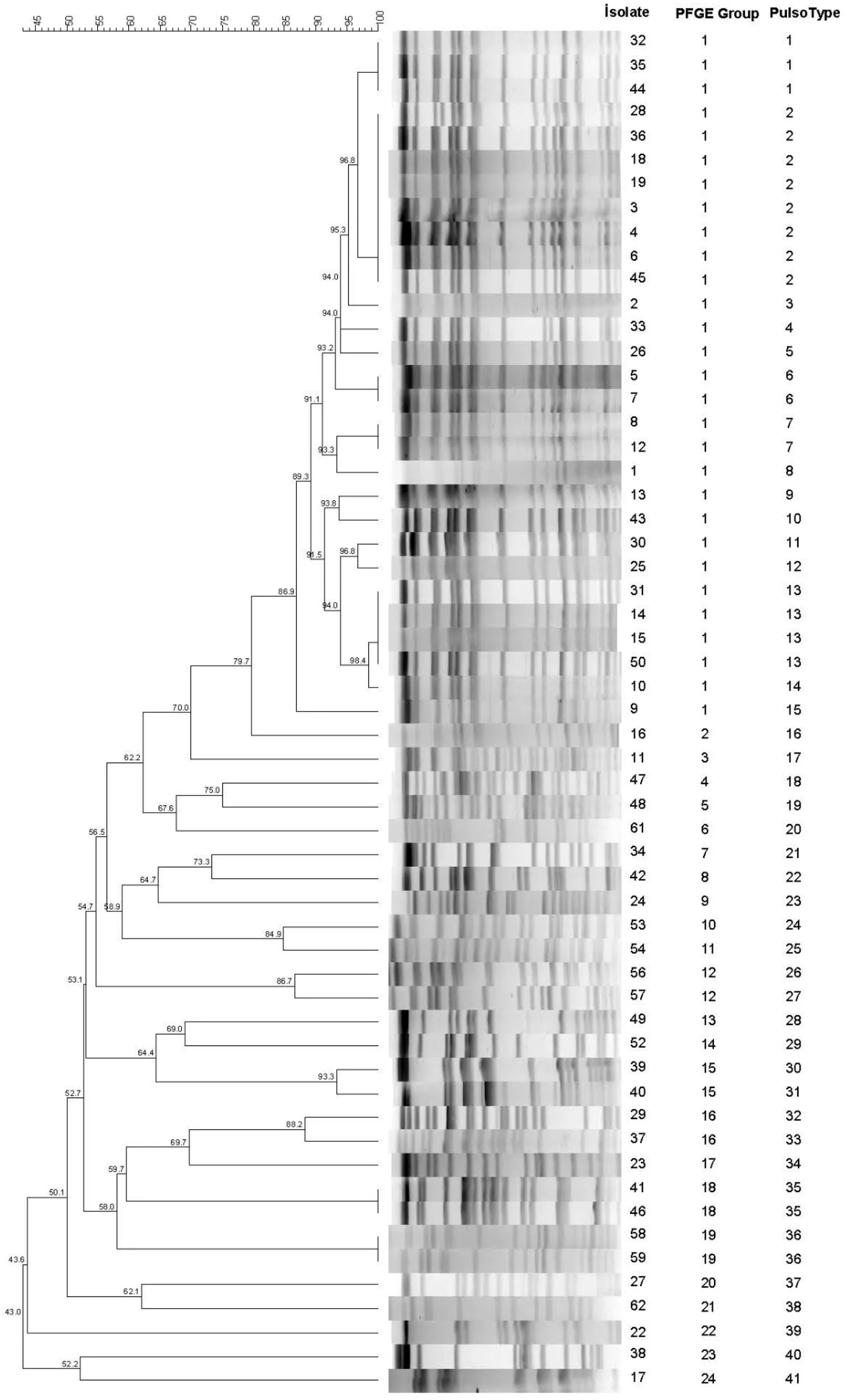
PFGE dendrogram of CRKP isolates.

The PFGE dendrogram showing the clonal relationships among the 57 isolates is presented in Figure 4. Comparison of PFGE profiles with conjugation results showed that conjugation-positive isolates were distributed across multiple PFGE groups and pulsotypes. Among the isolates included in the PFGE analysis, conjugative transfer was detected in isolates 35, 42, 43, 45, 47, 50, 56, 58, 59, 61, and 62. Four of these isolates (35, 43, 45, and 50) belonged to PFGE group 1 and represented different pulsotypes (PT1, PT10, PT2, and PT13, respectively). Isolates 58 and 59 belonged to PFGE group 19 and shared the same pulsotype (PT36).

In all conjugation-positive isolates, *blaOXA-48*, *blaTEM*, *blaCTX-M-1*, and *blaSHV* were detected in the corresponding transconjugants. Among the PMQR determinants, *aac(6′)-Ib-cr* was transferred only in isolate 42, whereas no other PMQR determinant was detected among the transferred genes. The PFGE group and pulsotype distributions of the conjugation-positive isolates are shown in Figure 4, while their donor and transconjugant phenotypic and genotypic profiles are presented in Table 2.

## 4. Discussion

The present study integrated antimicrobial susceptibility profiling, resistance-gene detection, conjugation experiments, plasmid replicon typing, and PFGE to investigate the molecular epidemiology of CRKP. The isolates exhibited extensive multidrug resistance, consistent with previous reports, providing the phenotypic context for the molecular findings [14,39,40,41]. Beyond the frequent detection of β-lactamase and carbapenemase determinants, the most distinctive findings were the high prevalence of *qnrD*, its absence among the recovered transconjugants, the consistent detection of *blaOXA-48*, *blaCTX-M-1*, *blaTEM*, and *blaSHV* in all 14 transconjugants, and the occurrence of conjugative transfer across multiple PFGE backgrounds. Together, these observations highlight the importance of examining resistance-gene distribution, experimentally detected transfer, and clonal relatedness as complementary aspects of CRKP epidemiology.

The resistance-gene profile of the present CRKP collection was characterized by the coexistence of multiple carbapenemase, β-lactamase, and PMQR determinants. Previous studies from Türkiye have similarly documented frequent detection of *blaOXA-48*, *blaSHV*, *blaCTX-M-1*, and *blaTEM*, although reported frequencies vary among centres and study populations [18,42]. Considerable geographical variation has also been reported internationally, particularly for *blaOXA-48* and *blaNDM*, while high frequencies of ESBL-associated determinants such as *blaSHV*, *blaCTX-M*, and *blaTEM* are common in multidrug-resistant *K. pneumoniae* populations [43,44,45,46]. In the present collection, the high frequencies of *blaSHV*, *blaCTX-M-1*, and *blaTEM*, together with the predominance of *blaOXA-48* and limited detection of *blaNDM*, are broadly consistent with the *blaOXA-48* centred molecular epidemiology previously described in Türkiye. The additional presence of *blaOXA-1* further illustrates the diversity and co-occurrence of β-lactamase determinants within this CRKP population. Although *blaOXA-48* was detected in 41 of the 62 CRKP isolates (66.1%), its detection does not account for carbapenem resistance in the entire collection. Carbapenem resistance in the remaining 21 isolates may involve other carbapenemases not detected by the PCR panel used, alterations in outer-membrane permeability, efflux, or combinations of resistance mechanisms. As these mechanisms were not comprehensively investigated in the present study, their individual contributions cannot be determined from our data. Variations in resistance-gene prevalence reported across studies may be attributable to differences in study population, specimen distribution, hospital setting, geographic region, sampling period, and molecular methodologies.

Among the PMQR determinants, *qnrD* represented one of the most distinctive findings of the study. Previous studies from Türkiye have reported *qnrA*, *qnrB*, *qnrS*, *aac(6′)-Ib-cr*, *qepA*, and *oqxAB* among quinolone-resistant *K. pneumoniae*, whereas studies that specifically included *qnrD* in their screening panels did not report its detection in clinical *Klebsiella* isolates [47,48]. Internationally, *qnrD* has generally been reported less frequently than other PMQR determinants [49,50]. Therefore, the high prevalence of sequence-confirmed *qnrD* in the present collection is epidemiologically notable and, to the best of our knowledge, represents the first report identified in our literature search of *qnrD* in clinical *K. pneumoniae* isolates from Türkiye. Importantly, *qnrD* was not detected in any transconjugant. This finding is consistent with reports indicating that *qnrD* may occur on small non-transmissible plasmids, particularly among Proteeae [51]. Thus, the high prevalence of *qnrD* among the donor isolates, together with its absence from the transconjugants, suggests a dissemination pattern distinct from that of the readily transferable β-lactam resistance determinants under the experimental conditions used. However, its precise genomic or plasmid location cannot be determined without direct sequencing-based localization.

Conjugation experiments provided direct functional evidence for the transferability of a substantial component of the β-lactam resistance background. Previous studies have demonstrated transferable *blaOXA-48* in *K. pneumoniae* [52,53,54]. In the present study, *blaTEM*, *blaSHV*, *blaCTX-M-1*, and *blaOXA-48* were consistently detected in all transconjugants, demonstrating a reproducibly transferable β-lactam resistance phenotype. In contrast, fluoroquinolone-resistance determinants, including *qnrD*, were largely not co-transferred, with *aac(6′)-Ib-cr* detected in only one transconjugant. The epidemiological significance of these findings therefore lies not simply in the multidrug-resistant phenotype of the donor population, but in the distinct transfer patterns exhibited by different resistance determinants. The 14/62 (22.6%) proportion of conjugation-positive isolates represents the percentage of donor isolates from which transconjugants were recovered under the experimental conditions used and should not be interpreted as a quantitative measure of conjugation frequency or transfer efficiency.

PBRT further characterized the plasmid replicon backgrounds associated with the transferable resistance phenotype. Previous studies from Türkiye have reported heterogeneous replicon profiles among CRKP isolates, including IncR, IncA/C, IncFIIK, and IncI1, while IncFIB has also been recognized among multidrug-resistant *K. pneumoniae* populations [5,42,55]. International studies have likewise reported IncFIB in combination with other replicon types, whereas *blaOXA-48* transfer has classically been associated with IncL plasmids [54,56]. Against this background, the predominance of IncFIB among the transconjugants in the present study is noteworthy. However, PBRT identifies replicon sequences rather than the physical location of individual resistance genes. Accordingly, IncFIB should be interpreted as the predominant associated replicon background of the transferable phenotype, not as direct evidence that the transferred β-lactamase genes were physically located on IncFIB plasmids. Confirmation of gene–plasmid linkage would require plasmid sequencing or other dedicated localization approaches.

PFGE analysis revealed a mixed epidemiological structure, with one predominant PFGE group occurring alongside numerous distinct groups and pulsotypes. Previous studies from Türkiye have reported both substantial PFGE clustering and marked clonal heterogeneity, while monoclonal *blaOXA-48* producing outbreaks have also been described [13,37,57,58]. Similar heterogeneity has been observed internationally [59,60]. Importantly, among the conjugation-positive isolates with interpretable PFGE profiles, transferable resistance was observed across multiple PFGE-defined genetic backgrounds rather than being restricted to a single predominant cluster. Taken together, these findings support the coexistence of PFGE-defined clonal relatedness and experimentally transferable resistance within the local CRKP population.

The present study has several limitations. The relatively small number of isolates, their retrospective collection from a single hospital, and the absence of detailed patient-level clinical metadata may limit the broader generalizability of the findings. PFGE profiles could not be obtained for five isolates, and the absence of MLST and whole-genome sequencing limited higher-resolution assessment of strain relatedness and lineage structure. In addition, although conjugation experiments and PBRT provided functional evidence of transferable resistance and characterized the associated replicon backgrounds, complete plasmid sequencing was not performed; therefore, the precise physical localization of individual resistance genes to specific plasmids could not be established. Quantitative conjugation frequencies were also not determined because endpoint transconjugant and donor/recipient counts were not available.

## 5. Conclusions

This study provides an integrated view of CRKP by combining phenotypic resistance, resistance-gene profiling, conjugation experiments, plasmid replicon typing, and PFGE-based clonal analysis. The findings indicate that the local CRKP population is characterized by a complex β-lactamase background dominated by *blaOXA-48* associated resistance, together with extensive multidrug resistance. The high prevalence of sequence-confirmed *qnrD*, which to the best of our knowledge represents the first report identified in our literature search in clinical *K. pneumoniae* isolates from Türkiye, is epidemiologically noteworthy; however, its precise genomic or plasmid location remains unresolved and requires sequencing-based confirmation. In contrast to its widespread occurrence among the clinical isolates, *qnrD* was not detected among the transferred determinants, whereas major β-lactamase genes were consistently recovered in the transconjugants. Conjugation experiments demonstrated the acquisition of β-lactam resistance phenotypes and the corresponding PCR-detected resistance determinants by the recipient under the experimental conditions used. However, determining the precise physical localization of these genes on individual plasmids would require plasmid sequencing or other dedicated localization approaches. Together with the PFGE evidence of clonal clustering and multiple genetic backgrounds, these findings are consistent with the coexistence of clonal relatedness and horizontally transferable resistance within the local CRKP population, without establishing the relative contribution of either process to their dissemination. Continued molecular surveillance, particularly incorporating whole-genome and complete plasmid sequencing, will be important for resolving the genetic platforms responsible for resistance dissemination and for monitoring the emergence and spread of clinically important CRKP lineages.

## Figures and Tables

**Table 1:** Distribution of resistance determinants among CRKP isolates (*n* = 62), with 95% confidence intervals.

Target Gene	Positive Isolates *n*/62	Prevalence (%)	95% CI
*oqxA*	62/62	100.0	94.2–100.0
*oqxB*	62/62	100.0	94.2–100.0
*blaSHV*	60/62	96.8	89.0–99.1
*blaCTX-M-1*	58/62	93.5	84.6–97.5
*blaTEM*	53/62	85.5	74.7–92.2
*aac(6′)-Ib-cr*	53/62	85.5	74.7–92.2
*qnrD*	43/62	69.4	57.0–79.4
*blaOXA-48*	41/62	66.1	53.7–76.7
*blaOXA-1*	29/62	46.8	34.9–59.0
*qnrS*	24/62	38.7	27.6–51.2
*blaNDM*	3/62	4.8	1.7–13.3
*qnrB*	3/62	4.8	1.7–13.3

Values are presented as number of positive isolates/total isolates tested, percentage, and 95% confidence interval (CI). Confidence intervals were calculated using the Wilson score method [38].

**Table 2:** Phenotypic and genotypic characteristics of conjugation-positive *K. pneumoniae* isolates and their corresponding transconjugants.

Donor Isolate	Corresponding Transconjugant	Associated Replicon Background	Donor Resistance Phenotype	Donor Resistance Genotype	Transconjugant Resistance Phenotype	Transconjugant Resistance Genotype
Kp35	TcKp35	IncY (+)	AM, AMC, CXM, CRO, ETP, MEM, IPM, CIP, AK, LEV, TZP, CAZ	*blaTEM*, *blaSHV*, *blaCTX-M-1*, *blaOXA-48*	AM, AMC, CXM, CRO, ETP, MEM, IPM, TZP, CAZ	*blaTEM*, *blaSHV*, *blaCTX-M-1*, *blaOXA-48*
Kp42	TcKp42	IncFIB (+), FrepB (+)	AM, AMC, CXM, CRO, ETP, MEM, IPM, CIP, AK, CN, LEV, TZP, CAZ	*blaTEM*, *blaSHV*, *blaCTX-M-1*, *blaOXA-48*, *aac(6′)-Ib-cr*	AM, AMC, CXM, CRO, ETP, MEM, IPM, CN, TZP, CAZ	*blaTEM*, *blaSHV*, *blaCTX-M-1*, *blaOXA-48*, *aac(6′)-Ib-cr*
Kp43	TcKp43	IncFIB (+)	AM, AMC, CXM, CRO, ETP, MEM, IPM, CIP, AK, LEV, TZP, CAZ	*blaTEM*, *blaSHV*, *blaCTX-M-1*, *blaOXA-48*	AM, AMC, CXM, CRO, ETP, MEM, IPM, TZP, CAZ	*blaTEM*, *blaSHV*, *blaCTX-M-1*, *blaOXA-48*
Kp45	TcKp45	IncFIB (+), FrepB (+)	AM, AMC, CXM, CRO, ETP, MEM, IPM, CIP, AK, LEV, TZP, CAZ	*blaTEM*, *blaSHV*, *blaCTX-M-1*, *blaOXA-48*	AM, AMC, CXM, CRO, ETP, MEM, IPM, TZP, CAZ	*blaTEM*, *blaSHV*, *blaCTX-M-1*, *blaOXA-48*
Kp47	TcKp47	IncFIB (+)	AM, AMC, CXM, ETP, MEM, IPM, CIP, AK, CN, LEV, TZP, CAZ	*blaTEM*, *blaSHV*, *blaCTX-M-1*, *blaOXA-48*	AM, AMC, CXM, ETP, MEM, IPM, TZP, CAZ	*blaTEM*, *blaSHV*, *blaCTX-M-1*, *blaOXA-48*
Kp50	TcKp50	IncFIB (+)	AM, AMC, CXM, ETP, MEM, IPM, CN, CIP, AK, LEV, TZP, CAZ	*blaTEM*, *blaSHV*, *blaCTX-M-1*, *blaOXA-48*	AM, AMC, CXM, ETP, MEM, IPM, TZP, CAZ	*blaTEM*, *blaSHV*, *blaCTX-M-1*, *blaOXA-48*
Kp51	TcKp51	IncFIB (+)	AM, AMC, CXM, ETP, MEM, IPM, CN, CIP, AK, LEV, TZP, CAZ	*blaTEM*, *blaSHV*, *blaCTX-M-1*, *blaOXA-48*	AM, AMC, CXM, ETP, MEM, IPM, TZP	*blaTEM*, *blaSHV*, *blaCTX-M-1*, *blaOXA-48*
Kp55	TcKp55	IncFIB (+)	AM, AMC, CXM, ETP, MEM, IPM, CN, CIP, AK, LEV, TZP, CAZ	*blaTEM*, *blaSHV*, *blaCTX-M-1*, *blaOXA-48*	AM, AMC, CXM, ETP, MEM, IPM, TZP	*blaTEM*, *blaSHV*, *blaCTX-M-1*, *blaOXA-48*
Kp56	TcKp56	IncFIB (+)	AM, AMC, CXM, CRO, ETP, MEM, IPM, CIP, AK, CN, LEV, TZP, CAZ	*blaTEM*, *blaSHV*, *blaCTX-M-1*, *blaOXA-48*	AM, AMC, CXM, CRO, ETP, MEM, IPM, TZP	*blaTEM*, *blaSHV*, *blaCTX-M-1*, *blaOXA-48*
Kp58	TcKp58	IncFIB (+)	AM, AMC, CXM, CRO, ETP, MEM, IPM, CIP, AK, CN, LEV, TZP, CAZ	*blaTEM*, *blaSHV*, *blaCTX-M-1*, *blaOXA-48*	AM, AMC, CXM, CRO, ETP, MEM, IPM, TZP	*blaTEM*, *blaSHV*, *blaCTX-M-1*, *blaOXA-48*
Kp59	TcKp59	IncFIB (+)	AM, AMC, CXM, ETP, MEM, IPM, CIP, AK, CN, LEV, TZP, CAZ	*blaTEM*, *blaSHV*, *blaCTX-M-1*, *blaOXA-48*	AM, AMC, CXM, ETP, MEM, IPM, TZP	*blaTEM*, *blaSHV*, *blaCTX-M-1*, *blaOXA-48*
Kp60	TcKp60	IncFIB (+)	AM, AMC, CXM, CRO, ETP, MEM, IPM, CIP, AK, LEV, TZP, CAZ	*blaTEM*, *blaSHV*, *blaCTX-M-1*, *blaOXA-48*	AM, AMC, CXM, CRO, ETP, MEM, IPM, TZP, CAZ	*blaTEM*, *blaSHV*, *blaCTX-M-1*, *blaOXA-48*
Kp61	TcKp61	IncFIB (+)	AM, AMC, CXM, CRO, ETP, MEM, IPM, CIP, AK, CN, LEV, TZP, CAZ	*blaTEM*, *blaSHV*, *blaCTX-M-1*, *blaOXA-48*	AM, AMC, CXM, CRO, ETP, MEM, IPM, TZP, CAZ	*blaTEM*, *blaSHV*, *blaCTX-M-1*, *blaOXA-48*
Kp62	TcKp62	IncFIB (+)	AM, AMC, CXM, CRO, ETP, MEM, IPM, CIP, AK, CN, LEV, TZP, CAZ	*blaTEM*, *blaSHV*, *blaCTX-M-1*, *blaOXA-48*	AM, AMC, CXM, CRO, ETP, MEM, IPM, TZP, CAZ	*blaTEM*, *blaSHV*, *blaCTX-M-1*, *blaOXA-48*

AM, ampicillin; AMC, amoxicillin/clavulanic acid; CXM, cefuroxime; CRO, ceftriaxone; ETP, ertapenem; MEM, meropenem; IPM, imipenem; CIP, ciprofloxacin; AK, amikacin; CN, gentamicin; LEV, levofloxacin; TZP, piperacillin/tazobactam; CAZ, ceftazidime. Antimicrobial resistance phenotypes were interpreted according to the EUCAST [15] criteria applied in this study. TcKp denotes the transconjugant corresponding to the indicated *K. pneumoniae* donor isolate. Quantitative conjugation frequency was not calculated because endpoint colony counts of transconjugants and donor/recipient cells were not performed.

## Data Availability

The data supporting the findings of this study are available from the corresponding author upon reasonable request.

## Supplementary Materials

The following supporting information can be downloaded at https://www.mdpi.com/article/10.3390/microorganisms14102181/s1, Table S1. Primer sequences used for the detection of resistance genes and plasmid replicon typing in *K. pneumoniae* isolates; Table S2. Gene-specific PCR cycling conditions used for the detection of carbapenemase, PMQR, and extended-spectrum β-lactamase (ESBL) genes in carbapenem-resistant *K. pneumoniae* isolates; Table S3. Antimicrobial resistance profiles and distribution of resistance determinants among the 62 carbapenem-resistant *K. pneumoniae* clinical isolates; Table S4. Minimum inhibitory concentrations (MICs) of ertapenem, imipenem, and meropenem in the 62 carbapenem-resistant *K. pneumoniae* clinical isolates.
