# Supplementary material for: Clonal Relatedness and Transferable Resistance in Carbapenem-Resistant Klebsiella pneumoniae: An Integrated Molecular Epidemiological Study
Source: Microorganisms. 2026 Sep 29;14(10):2181. doi: 10.3390/microorganisms14102181 (PMC13707725; doi:10.3390/microorganisms14102181)
Supplement: Supplementary file 1 [file microorganisms-14-02181-s001.zip › Supplementary Table S1.pdf]

Suplemantary Table S1. Primer sequences used for detection of resistance genes and plasmid replicon typing in *K. pneumoniae* isolates.

Gene Category	Target Gene	Primer Sequence (5'→3')	Amplicon Size (bp)	Reference
Carbapenemase	*SHV*	**F:** ATGCGTTATATTCGCCTGTG<br>**R:** TTAGCGTTGCCAGTGCTC	843	[1]
	TEM	**F:** AGTATTCAACATTTYCGTGT<br>**R:** TAATCAGTGAGGCACCTATCTC	847	[2]
	CTX-M1	**F:** GCGTGATACCACTTCACCTC<br>**R:** TGAAGTAAGTGACCAGAATC	260	
	CTX-M2	**F:** TGATACCACCACGCCGCTC<br>**R:** TATTGCATCAGAAACCGTGGG	341	
	GES	**F:** ATGCGCTTCATTCACGCAC<br>**R:** CTATTTGTCCGTGCTCAGGA	863	
	KPC	**F:** ATGTCACTGTATCGCCGTCT<br>**R:** TTTTCAGAGCCTTACTGCCC	893	[3]
	IMP	**F:** CATGGTTTGGTGGTTCTTGT<br>**R:** ATAATTTGGCGGACTTTGGC	488	[4]
	VIM	**F:** ATTGGTCTATTTGACCGCGTC<br>**R:** TGCTACTCAACGACTGAGCG	780	
	NDM-1	**F:** TGGAATTGCCCAATATTATGC<br>**R:** TCAGCGCAGCTTGTCGGCCATGC	621	[5]
	VEB	**F:** GATAGGAGTACAGACATATG<br>**R:** TTTATTCAAATAGTAATTCCACG	914	[6]
	PER-1	**F:** ATGAATGTCATCACAAAATG<br>**R:** TCAATCCGGACTCACT	927	
	OXA-23	**F:** GATCGGATTGGAGAACCAGA<br>**R:** ATTTCTGACCGCATTTCCAT	501	[7]
	OXA-58	**F:** AAGTATTGGGGCTTGTGCTG<br>**R:** CCCCTCTGCGCTCTACATAC	599	
	OXA-51	**F:** TAATGCTTTGATCGGCCTTG<br>**R:** TGGATTGCACTTCATCTTGG	353	
	OXA-40	**F:** GGTTAGTTGGCCCCCTTAAA<br>**R:** AGTTGAGCGAAAAGGGGATT	246	
	OXA-48	**F:** TTGGTGGCATCGATTATCGG<br>**R:** GAGCACTTCTTTTGTGATGGC	743	[8]
	OXA-1	**F:** TTTTCTGTTGTTTGGGTTTT<br>**R:** TTTCTTGGCTTTTATGCTTG	427	[9]
Quinolone (PMQR)	*qnrA*	**F:** GCCCGCTTCTACAATCAAGT<br>**R:** GGCAGCACTATTACTCCCAAG	347	[10]
	qnrS	**F:** TCGGCACCACAACTTTTCAC<br>**R:** TCACACGCACGGAACTCTAT	255	
	qnrB	**F:** TGGCAAAAAATTGAACAGAA<br>**R:** GAGCAACGATCGCCTGGTAG	594	[11]
	qnrC	**F:** GGGTTGTACATTTATTGAATC<br>**R:** TCCACTTTACGAGGTTCT	447	[12]
	qnrD	**F:** CGAGATCAATTTACGGGGAATA<br>**R:** AACAAGCTGAAGCGCCTG	582	[13]

Gene Category	Target Gene	Primer Sequence (5’→3’)	Amplicon Size (bp)	Reference
	qepA	**F:** CTGCAGGTACTGCGTCATG<br>**R:** CGTGTTGCTGGAGTTCTTC	403	[14]
	oqxA	**F:** GACAGCGTCGCACAGAATG<br>**R:** GGAGACGAGGTTGGTATGGA	339	
	oqxB	**F:** CGAAGAAAGACCTCCCTACCC<br>**R:** CGCCGCCAATGAGATACA	240	
	aac(6’)-Ib-cr	**F:** TTGCGATGCTCTATGAGTGGCTA<br>**R:** CTCGAATGCCTGGCGTGTTT	482	[15]
**Class 1 Integron**	Variable gene-cassette region	5’-CS: GGCATCCAAGCAGCAAG<br>3’-CS: AAGCAGACTTGACCTGA	Variable	[16]
**Class 2 Integron**	Variable gene-cassette region	hep51: GATGCCATCGCAAGTACGAG<br>hep74: GGGATCCCGGACGGATGCACGATTTGTA	Variable	[17]
**Plasmid Replicon Typing (PBRT)**	*HI1*	**F:** GGAGCGATGGATTACTTCAGTAC<br>**R:** TGCCGTTTCACCTCGTGAGTA	471	[18]
	HI2	**F:** TTTCTCCTGAGTCACCTGTTAACAC<br>**R:** GGCTCACTACCGTTGTCATCCT	644	
	I1	**F:** CGAAAGCCGGACGGCAGAA<br>**R:** TCGTCGTTCCGCCAAGTTCGT	139	
	X	**F:** AACCTTAGAGGCTATTTAAGTTGCTGAT<br>**R:** TGAGAGTCAATTTTTATCTCATGTTTTAGC	376	
	L/M	**F:** GGATGAAAACTATCAGCATCTGAAG<br>**R:** CTGCAGGGGCGATTCTTTAGG	785	
	N	**F:** GTCTAACGAGCTTACCGAAG<br>**R:** GTTTCAACTCTGCCAAGTTC	559	
	FIA	**F:** CCATGCTGGTTCTAGAGAAGGTG<br>**R:** GTATATCCTTACTGGCTTCCGCAG	462	
	FIB	**F:** GGAGTTCTGACACACGATTTTCTG<br>**R:** CTCCCGTCGCTTCAGGGCATT	702	
	W	**F:** CCTAAGAACAACAAAGCCCCCG<br>**R:** GGTGCGCGGCATAGAACCGT	242	
	Y	**F:** AATTCAAACAACACTGTGCAGCCTG<br>**R:** GCGAGAATGGACGATTACAAAACTTT	765	
	P	**F:** CTATGGCCCTGCAAACGCGCCAGAAA<br>**R:** TCACGCGCCAGGGCGCAGCC	534	
	FIC	**F:** GTGAACTGGCAGATGAGGAAGG<br>**R:** TTCTCCTCGTCGCCAAACTAGAT	262	
	A/C	**F:** GAGAACCAAAGACAAAGACCTGGA<br>**R:** ACGACAAACCTGAATTGCCTCCTT	465	
	T	**F:** TTGGCCTGTTTGTGCCTAAACCAT<br>**R:** CGTTGATTACACTTAGCTTTGGAC	750	
	FIIs	**F:** CTGTCGTAAGCTGATGGC<br>**R:** CTCTGCCACAAACTTCAGC	270	
	B/O	**F:** GCGGTCCGGAAAGCCAGAAAAC<br>**R:** TCTGCGTTCCGCCAAGTTCGA	159	
	K/B-K	**F:** GCGGTCCGGAAAGCCAGAAAAC	160	

Gene Category	Target Gene	Primer Sequence (5'→3')	Amplicon Size (bp)	Reference
		**R:** TCTTTCACGAGCCCGCCAAA		
	FrepB	**F:** TGATCGTTTAAGGAATTTTG<br>**R:** GAAGATCAGTCACACCATCC	270	

1. Hanson, N.D.; Moland, E.S.; Hossain, A.; Neville, S.A.; Gosbell, I.B.; Thomson, K.S. Unusual Salmonella enterica serotype Typhimurium isolate producing CMY-7, SHV-9 and OXA-30 β-lactamases. *J. Antimicrob. Chemother.* **2002**, *49*, 1011–1014.
2. Iraz, M.; Özad Düzgün, A.; Sandallı, C.; Doymaz, M.Z.; Akkoyunlu, Y.; Saral, A.; Peleg, A.Y.; Özgümüş, O.B.; Beriş, F.Ş.; Karaoğlu, H. Distribution of β-lactamase genes among carbapenem-resistant Klebsiella pneumoniae strains isolated from patients in Turkey. *Ann. Lab. Med.* **2015**, *35*, 595–601.
3. Schechner, V.; Straus-Robinson, K.; Schwartz, D.; Pfeffer, I.; Tarabeia, J.; Moskovich, R.; Chmelnitsky, I.; Schwaber, M.J.; Carmeli, Y.; Navon-Venezia, S. Evaluation of PCR-based testing for surveillance of KPC-producing carbapenem-resistant members of the Enterobacteriaceae family. *J. Clin. Microbiol.* **2009**, *47*, 3261–3265.
4. Jeon, B.-C.; Jeong, S.H.; Bae, I.K.; Kwon, S.B.; Lee, K.; Young, D.; Lee, J.H.; Song, J.S.; Lee, S.H. Investigation of a nosocomial outbreak of imipenem-resistant Acinetobacter baumannii producing the OXA-23 β-lactamase in Korea. *J. Clin. Microbiol.* **2005**, *43*, 2241–2245.
5. Poirel, L.; Walsh, T.R.; Cuvillier, V.; Nordmann, P. Multiplex PCR for detection of acquired carbapenemase genes. *Diagn. Microbiol. Infect. Dis.* **2011**, *70*, 119–123.
6. Kiratisin, P.; Apisarnthanarak, A.; Laesripa, C.; Saifon, P. Molecular characterization and epidemiology of extended-spectrum-β-lactamase-producing Escherichia coli and Klebsiella pneumoniae isolates causing health care-associated infection in Thailand, where the CTX-M family is endemic. *Antimicrob. Agents Chemother.* **2008**, *52*, 2818–2824.
7. Woodford, N.; Ellington, M.J.; Coelho, J.M.; Turton, J.F.; Ward, M.E.; Brown, S.; Amyes, S.G.; Livermore, D.M. Multiplex PCR for genes encoding prevalent OXA carbapenemases in Acinetobacter spp. *Int. J. Antimicrob. Agents* **2006**, *27*, 351–353.
8. Poirel, L.; Potron, A.; Nordmann, P. OXA-48-like carbapenemases: the phantom menace. *J. Antimicrob. Chemother.* **2012**, *67*, 1597–1606.
9. Coque, T.M.; Novais, Â.; Carattoli, A.; Poirel, L.; Pitout, J.; Peixe, L.; Baquero, F.; Cantón, R.; Nordmann, P. Dissemination of clonally related Escherichia coli strains expressing extended-spectrum β-lactamase CTX-M-15. *Emerg. Infect. Dis.* **2008**, *14*, 195.
10. Hamed, S.M.; Aboshanab, K.M.; El-Mahallawy, H.A.; Helmy, M.M.; Ashour, M.S.; Elkhatib, W.F. Plasmid-mediated quinolone resistance in gram-negative pathogens isolated from cancer patients in Egypt. *Microbial drug resistance* **2018**, *24*, 1316–1325.
11. Bouchakour, M.; Zerouali, K.; Claude, J.D.P.G.; Amarouch, H.; El Mdaghri, N.; Courvalin, P.; Timinouni, M. Plasmid-mediated quinolone resistance in expanded spectrum beta lactamase producing enterobacteriaceae in Morocco. *The Journal of Infection in Developing Countries* **2010**, *4*, 779–803.
12. Wang, A.; Yang, Y.; Lu, Q.; Wang, Y.; Chen, Y.; Deng, L.; Ding, H.; Deng, Q.; Wang, L.; Shen, X. Occurrence of qnr-positive clinical isolates in Klebsiella pneumoniae producing ESBL or AmpC-type β-lactamase from five pediatric hospitals in China. *FEMS Microbiol. Lett.* **2008**, *283*, 112–116.
13. Pribul, B.R.; Festivo, M.L.; Souza, M.M.S.d.; Rodrigues, D.d.P. Characterization of quinolone resistance in Salmonella spp. isolates from food products and human samples in Brazil. *Braz. J. Microbiol.* **2016**, *47*, 196–201.
14. Goudarzi, M.; Azad, M.; Seyedjavadi, S.S. Prevalence of Plasmid-Mediated Quinolone Resistance Determinants and OqxAB Efflux Pumps among Extended-Spectrum β-Lactamase Producing Klebsiella pneumoniae Isolated from Patients with Nosocomial Urinary Tract Infection in Tehran, Iran. *Scientifica* **2015**, *2015*, 518167.
15. Park, C.H.; Robicsek, A.; Jacoby, G.A.; Sahm, D.; Hooper, D.C. Prevalence in the United States of aac (6′)-Ib-cr encoding a ciprofloxacin-modifying enzyme. *Antimicrob. Agents Chemother.* **2006**, *50*, 3953–3955.
16. Levesque, C.; Piche, L.; Larose, C.; Roy, P.H. PCR mapping of integrons reveals several novel combinations of resistance genes. *Antimicrob. Agents Chemother.* **1995**, *39*, 185–191.
17. White, P.A.; McIver, C.J.; Rawlinson, W.D. Integrons and gene cassettes in the Enterobacteriaceae. *Antimicrob. Agents Chemother.* **2001**, *45*, 2658–2661.
18. Carattoli, A.; Bertini, A.; Villa, L.; Falbo, V.; Hopkins, K.L.; Threlfall, E.J. Identification of plasmids by PCR-based replicon typing. *J. Microbiol. Methods* **2005**, *63*, 219–228.
