# Supplementary material for: Clonal Relatedness and Transferable Resistance in Carbapenem-Resistant Klebsiella pneumoniae: An Integrated Molecular Epidemiological Study
Source: Microorganisms. 2026 Sep 29;14(10):2181. doi: 10.3390/microorganisms14102181 (PMC13707725; doi:10.3390/microorganisms14102181)
Supplement: Supplementary file 1 [file microorganisms-14-02181-s001.zip › Supplementary Table S2.pdf]

Suplemantary Table S2: Gene-specific PCR cycling conditions used for the detection of carbapenemase, PMQR and extended-spectrum β-lactamase (ESBL) genes in carbapenem-resistant *Klebsiella pneumoniae* isolates.

Target gene	Initial denaturation	Cycles	Denaturation	Annealing	Extension	Final extension	References
SHV	95°C 3 min	35×	95°C 50 s	56°C 50 s	72°C 1 min	72°C 5 min	[1]
TEM			95°C 50 s	50°C 50 s	72°C 1 min		
CTX-M1			95°C 45 s	54°C 45 s	72°C 45 s		
CTX-M2			95°C 45 s	59°C 45 s	72°C 45 s		
GES			95°C 50 s	57°C 50 s	72°C 1 min		
KPC			95°C 50 s	58°C 50 s	72°C 50 s		
IMP			95°C 45 s	56°C 45 s	72°C 45 s		
VIM			95°C 50 s	59°C 50 s	72°C 50 s		
NDM-1			95°C 50 s	58°C 50 s	72°C 50 s		
VEB			95°C 50 s	55°C 50 s	72°C 50 s		
PER-1			95°C 50 s	48°C 50 s	72°C 50 s		
OXA-23			95°C 45 s	54°C 45 s	72°C 45 s		
OXA-58			95°C 50 s	58°C 50 s	72°C 50 s		
OXA-51			95°C 45 s	56°C 45 s	72°C 45 s		
OXA-40			95°C 45 s	58°C 45 s	72°C 45 s		
OXA-48			95°C 50 s	58°C 50 s	72°C 50 s		
OXA-1			95°C 50 s	50°C 50 s	72°C 50 s		
qnrC			95°C 45 s	58°C 45 s	72°C 45 s		
qepA			95°C 45 s	58°C 45 s	72°C 45 s		
qnrA, *qnrB*, *qnrS* *aac(6')-Ib-cr*	95°C 5 min	30×	95°C 45 s	56°C 30 s	65°C 2.5 min	65°C 10 min	This Study
oqxA, oqxB, qnrD				58°C 30 s	65°C 2 min		This Study

Target(s)	Positive controls
blaOXA-1, blaOXA-48, blaCTX-M-1, blaSHV, blaTEM	DNA from previously characterized *Acinetobacter baumannii* isolates obtained from the culture collection of the Department of Medical Biology, Kırşehir Ahi Evran University Faculty of Medicine [1]
oqxA, oqxB	DNA from previously characterized *Acinetobacter baumannii* isolates obtained from the culture collection of the Department of Medical Biology, Kırşehir Ahi Evran University Faculty of Medicine [2]
qnrD	Previously characterized *Proteus mirabilis* DNA, Department of Medical Biology, Kırşehir Ahi Evran University Faculty of Medicine (provided by Prof. Dr. Elif Sevim). The identity of the *qnrD* PCR product was additionally confirmed by bidirectional Sanger sequencing of the amplicon obtained from one representative *qnrD*-positive *K. pneumoniae* isolate.
qnrA, qnrB, qnrS, aac(6')-Ib-cr	Previously characterized positive-control DNA, Public Health General Directorate (HSGM), Türkiye (provided by Dr. Zekiye Bakkaloğlu)
qepA	Yeliz Tanrıverdi Çaycı (Ondokuz Mayıs University, Faculty of Medicine, Department of Medical Microbiyology, Samsun, Türkiye) for sharing *qepA* positive *E. coli* strain.

1. Öztürk, C. Comprehensive Molecular Characterization of Extensively Drug-Resistant Acinetobacter baumannii Isolated from Intensive Care Unit Patients: Carbapenemase Genes, Plasmid-Mediated Resistance Determinants, and PFGE-Based Clonal Analysis. *Pharmaceuticals* **2026**, *19*, 862.
2. Abdalh, D.N.; Öztürk, C.; Sevim, E.; Akyol, R. Investigation of plasmid mediated quinolone resistance genes in quinolone-resistant Acinetobacter baumannii isolates. *Microbes and Infectious Diseases* **2026**, *7*, 2193–2201, doi:10.21608/mid.2025.415693.3131.
