# Supplementary material for: Clonal Relatedness and Transferable Resistance in Carbapenem-Resistant Klebsiella pneumoniae: An Integrated Molecular Epidemiological Study
Source: Microorganisms. 2026 Sep 29;14(10):2181. doi: 10.3390/microorganisms14102181 (PMC13707725; doi:10.3390/microorganisms14102181)
Supplement: Supplementary file 1 [file microorganisms-14-02181-s001.zip › Supplementary Table S3.pdf]

Supplementary Table S3: Antimicrobial resistance profiles and distribution of resistance determinants among the 62 carbapenem-resistant Klebsiella pneumoniae clinical isolates.

		**Antimicrobial susceptibility**														**β-lactamase / carbapenemase genes**																	**Quinolone resistance determinants**								
**Isolate No.**	**Specimen type**	AM	AMC	CXM	CRO	ETP	MEM	IMP	CIP	AK	CN	LEV	TPZ	CAZ	SXT	*SHV*	*TEM*	*CTX- M1*	*CTX-M2*	*GES*	*KPC*	*IMP*	*VIM*	*NDM*	*VEB*	*PER1*	*OXA-1*	*OXA-23*	*OXA-58*	*OXA-51*	*OXA-48*	*OXA-40*	*qnrA*	*qnrB*	*qnrC*	*qnrD*	*qnrS*	*aac (6)-Ib-cr*	*qepA*	*oqxA*	*oqxB*
Kp1	BC	R	R	R	R	R	R	R	R	R	R	R	R	R	R	+	+	+	ø	ø	ø	ø	ø	ø	ø	ø	+	ø	ø	ø	+	ø	ø	ø	ø	ø	ø	+	ø	+	+
Kp2	BC	R	R	R	R	R	R	R	R	R	R	R	R	R	R	+	+	+	ø	ø	ø	ø	ø	ø	ø	ø	+	ø	ø	ø	+	ø	ø	ø	ø	ø	ø	+	ø	+	+
Kp3	WC	R	R	R	R	R	R	R	R	R	R	R	R	R	R	+	+	+	ø	ø	ø	ø	ø	ø	ø	ø	+	ø	ø	ø	+	ø	ø	ø	ø	ø	ø	+	ø	+	+
Kp4	U	R	R	R	S	R	R	R	R	R	R	R	R	R	R	+	+	+	ø	ø	ø	ø	ø	ø	ø	ø	ø	ø	ø	ø	+	ø	ø	ø	ø	ø	ø	+	ø	+	+
Kp5	SPT	R	R	R	R	R	R	R	R	R	R	R	R	R	R	+	+	+	ø	ø	ø	ø	ø	ø	ø	ø	+	ø	ø	ø	+	ø	ø	ø	ø	ø	ø	+	ø	+	+
Kp6	WC	R	R	R	R	R	R	R	R	R	R	R	R	R	R	+	+	+	ø	ø	ø	ø	ø	ø	ø	ø	ø	ø	ø	ø	+	ø	ø	ø	ø	ø	ø	+	ø	+	+
Kp7	U	R	R	R	R	R	R	R	R	R	R	R	R	R	R	+	+	+	ø	ø	ø	ø	ø	ø	ø	ø	+	ø	ø	ø	+	ø	ø	ø	ø	ø	ø	+	ø	+	+
Kp8	U	R	R	R	R	R	R	R	R	R	R	R	R	R	R	+	+	+	ø	ø	ø	ø	ø	ø	ø	ø	+	ø	ø	ø	+	ø	ø	ø	ø	ø	ø	+	ø	+	+
Kp9	U	R	R	R	R	R	R	R	R	R	R	R	R	R	R	+	+	+	ø	ø	ø	ø	ø	ø	ø	ø	+	ø	ø	ø	+	ø	ø	ø	ø	ø	ø	+	ø	+	+
Kp10	U	R	R	R	R	R	R	R	R	R	R	R	R	R	S	+	+	+	ø	ø	ø	ø	ø	ø	ø	ø	ø	ø	ø	ø	+	ø	ø	ø	ø	ø	ø	+	ø	+	+
Kp11	BC	R	R	R	R	R	R	R	R	R	R	R	R	R	R	+	+	+	ø	ø	ø	ø	ø	ø	ø	ø	ø	ø	ø	ø	+	ø	ø	ø	ø	ø	ø	+	ø	+	+
Kp12	BC	R	R	R	R	R	R	R	R	R	R	R	R	R	R	+	+	+	ø	ø	ø	ø	ø	ø	ø	ø	+	ø	ø	ø	+	ø	ø	ø	ø	ø	ø	+	ø	+	+
Kp13	U	R	R	R	R	R	R	R	R	R	R	R	R	R	R	+	+	+	ø	ø	ø	ø	ø	ø	ø	ø	+	ø	ø	ø	+	ø	ø	ø	ø	ø	ø	+	ø	+	+
Kp14	BC	R	R	R	R	R	R	R	R	R	R	R	R	R	R	+	+	+	ø	ø	ø	ø	ø	ø	ø	ø	+	ø	ø	ø	+	ø	ø	ø	ø	ø	ø	+	ø	+	+
Kp15	U	R	R	R	R	R	R	R	R	R	R	R	R	R	R	+	+	+	ø	ø	ø	ø	ø	ø	ø	ø	ø	ø	ø	ø	+	ø	ø	ø	ø	ø	ø	+	ø	+	+
Kp16	U	R	R	R	R	R	R	R	R	R	R	R	R	R	R	+	+	+	ø	ø	ø	ø	ø	ø	ø	ø	+	ø	ø	ø	+	ø	ø	ø	ø	ø	ø	+	ø	+	+
Kp17	U	R	R	R	R	R	R	R	R	R	R	R	R	R	R	+	+	+	ø	ø	ø	ø	ø	ø	ø	ø	+	ø	ø	ø	+	ø	ø	ø	ø	ø	ø	+	ø	+	+
Kp18	BC	R	R	R	R	R	R	R	R	R	R	R	R	R	R	+	+	+	ø	ø	ø	ø	ø	ø	ø	ø	+	ø	ø	ø	+	ø	ø	ø	ø	ø	ø	+	ø	+	+
Kp19	SPT	R	S	R	S	R	R	R	R	S	S	R	S	S	S	+	+	+	ø	ø	ø	ø	ø	ø	ø	ø	ø	ø	ø	ø	+	ø	ø	ø	ø	ø	ø	+	ø	+	+
Kp20	U	R	R	R	S	R	R	R	R	S	S	R	S	R	R	+	+	+	ø	ø	ø	ø	ø	ø	ø	ø	ø	ø	ø	ø	ø	ø	ø	ø	ø	+	ø	ø	ø	+	+
Kp21	U	R	R	R	R	R	R	R	R	S	S	R	S	R	S	+	+	+	ø	ø	ø	ø	ø	ø	ø	ø	ø	ø	ø	ø	+	ø	ø	ø	ø	+	ø	+	ø	+	+
Kp22	BC	R	R	R	R	R	R	R	R	R	R	R	R	R	R	+	+	+	ø	ø	ø	ø	ø	ø	ø	ø	ø	ø	ø	ø	ø	ø	ø	ø	ø	+	ø	ø	ø	+	+
Kp23	BC	R	R	R	R	R	R	R	R	R	R	R	R	R	R	+	ø	ø	ø	ø	ø	ø	ø	ø	ø	ø	ø	ø	ø	ø	ø	ø	ø	ø	ø	+	ø	ø	ø	+	+
Kp24	U	R	S	S	S	R	R	R	R	S	S	R	S	S	S	ø	ø	+	ø	ø	ø	ø	ø	ø	ø	ø	ø	ø	ø	ø	ø	ø	ø	ø	ø	+	ø	+	ø	+	+
Kp25	U	R	S	S	S	R	R	R	R	S	S	R	S	S	S	+	+	+	ø	ø	ø	ø	ø	ø	ø	ø	ø	ø	ø	ø	+	ø	ø	ø	ø	+	ø	+	ø	+	+
Kp26	U	R	R	R	R	R	R	R	R	R	R	R	R	s	R	+	+	+	ø	ø	ø	ø	ø	ø	ø	ø	ø	ø	ø	ø	+	ø	ø	ø	ø	+	ø	+	ø	+	+
Kp27	U	R	R	R	R	R	R	R	R	R	R	R	R	R	R	+	ø	ø	ø	ø	ø	ø	ø	ø	ø	ø	ø	ø	ø	ø	ø	ø	ø	+	ø	+	ø	ø	ø	+	+
Kp28	U	R	R	R	R	R	R	R	R	S	R	R	R	R	R	+	+	+	ø	ø	ø	ø	ø	ø	ø	ø	ø	ø	ø	ø	ø	ø	ø	ø	ø	+	ø	+	ø	+	+

Kp29	BC	R	R	S	S	R	R	R	R	R	R	R	R	R	R	+	+	+	ø	ø	ø	ø	ø	+	ø	ø	+	ø	ø	ø	ø	ø	ø	+	ø	+	ø	+	ø	+	+
Kp30	U	R	R	R	R	R	R	R	R	R	R	R	R	R	R	+	+	+	ø	ø	ø	ø	ø	ø	ø	ø	ø	ø	ø	ø	+	ø	ø	ø	ø	+	ø	+	ø	+	+
Kp31	U	R	R	R	R	R	R	R	R	R	R	R	R	R	R	+	+	+	ø	ø	ø	ø	ø	ø	ø	ø	ø	ø	ø	ø	+	ø	ø	ø	ø	+	ø	+	ø	+	+
Kp32	U	R	R	R	R	R	R	R	R	S	S	R	R	R	S	+	+	+	ø	ø	ø	ø	ø	ø	ø	ø	ø	ø	ø	ø	+	ø	ø	ø	ø	+	+	+	ø	+	+
Kp33	U	R	S	S	S	R	R	R	R	S	S	R	S	S	S	+	+	+	ø	ø	ø	ø	ø	ø	ø	ø	ø	ø	ø	ø	ø	ø	ø	ø	ø	+	ø	+	ø	+	+
Kp34	U	R	R	R	R	R	R	R	R	S	S	R	R	s	S	+	+	+	ø	ø	ø	ø	ø	ø	ø	ø	ø	ø	ø	ø	ø	ø	ø	ø	ø	+	+	+	ø	+	+
Kp35	U	R	R	R	R	R	R	R	R	S	S	R	R	S	S	+	+	+	ø	ø	ø	ø	ø	ø	ø	ø	+	ø	ø	ø	+	ø	ø	ø	ø	+	+	+	ø	+	+
Kp36	BC	R	s	R	S	R	R	R	R	S	S	R	S	S	S	+	+	+	ø	ø	ø	ø	ø	ø	ø	ø	+	ø	ø	ø	+	ø	ø	ø	ø	+	+	+	ø	+	+
Kp37	U	R	S	S	S	R	R	R	R	S	S	R	S	S	S	+	ø	ø	ø	ø	ø	ø	ø	ø	ø	ø	ø	ø	ø	ø	ø	ø	ø	ø	ø	+	ø	ø	ø	+	+
Kp38	U	R	R	R	S	R	R	R	R	R	S	R	R	s	S	ø	ø	ø	ø	ø	ø	ø	ø	ø	ø	ø	ø	ø	ø	ø	ø	ø	ø	ø	ø	+	+	+	ø	+	+
Kp39	U	R	R	R	R	R	R	R	R	R	R	R	R	R	R	+	+	+	ø	ø	ø	ø	ø	ø	ø	ø	ø	ø	ø	ø	ø	ø	ø	ø	ø	+	+	ø	ø	+	+
Kp40	U	R	R	S	S	R	R	R	R	S	S	R	R	S	S	+	ø	+	ø	ø	ø	ø	ø	ø	ø	ø	ø	ø	ø	ø	ø	ø	ø	ø	ø	+	ø	ø	ø	+	+
Kp41	U	R	R	R	R	R	R	R	R	S	R	R	R	R	R	+	+	+	ø	ø	ø	ø	ø	+	ø	ø	+	ø	ø	ø	ø	ø	ø	ø	ø	+	+	+	ø	+	+
Kp42	U	R	S	S	S	R	R	R	R	S	S	R	S	S	S	+	+	+	ø	ø	ø	ø	ø	ø	ø	ø	+	ø	ø	ø	ø	ø	ø	+	ø	+	ø	+	ø	+	+
Kp43	U	R	S	S	S	R	R	R	R	S	S	R	S	S	S	+	+	+	ø	ø	ø	ø	ø	ø	ø	ø	+	ø	ø	ø	+	ø	ø	ø	ø	+	+	+	ø	+	+
Kp44	U	R	R	R	R	R	R	R	R	R	R	R	R	R	R	+	+	+	ø	ø	ø	ø	ø	ø	ø	ø	+	ø	ø	ø	ø	ø	ø	ø	ø	+	ø	+	ø	+	+
Kp45	U	R	R	R	R	R	R	R	R	R	S	R	R	R	S	+	+	+	ø	ø	ø	ø	ø	ø	ø	ø	+	ø	ø	ø	+	ø	ø	ø	ø	+	+	+	ø	+	+
Kp46	U	R	S	S	S	R	R	R	R	S	S	R	S	S	R	+	+	+	ø	ø	ø	ø	ø	+	ø	ø	+	ø	ø	ø	ø	ø	ø	ø	ø	+	+	+	ø	+	+
Kp47	U	R	S	S	R	R	R	R	R	R	R	R	S	S	R	+	ø	+	ø	ø	ø	ø	ø	ø	ø	ø	+	ø	ø	ø	+	ø	ø	ø	ø	+	+	+	ø	+	+
Kp48	U	R	R	R	R	R	R	R	R	S	R	R	R	R	R	+	+	+	ø	ø	ø	ø	ø	ø	ø	ø	ø	ø	ø	ø	ø	ø	ø	ø	ø	+	+	+	ø	+	+
Kp49	BC	R	R	R	R	R	R	R	R	R	R	R	R	R	R	+	ø	+	ø	ø	ø	ø	ø	ø	ø	ø	ø	ø	ø	ø	ø	ø	ø	ø	ø	+	+	+	ø	+	+
Kp50	BC	R	R	R	R	R	R	R	R	R	R	R	R	R	R	+	+	+	ø	ø	ø	ø	ø	ø	ø	ø	+	ø	ø	ø	+	ø	ø	ø	ø	+	+	+	ø	+	+
Kp51	U	R	R	R	R	R	R	R	R	R	R	R	R	R	R	+	+	+	ø	ø	ø	ø	ø	ø	ø	ø	ø	ø	ø	ø	+	ø	ø	ø	ø	+	+	+	ø	+	+
Kp52	WC	R	R	R	R	R	R	R	R	R	R	R	R	R	R	+	ø	+	ø	ø	ø	ø	ø	ø	ø	ø	ø	ø	ø	ø	ø	ø	ø	ø	ø	+	ø	ø	ø	+	+
Kp53	BC	R	R	R	R	R	R	R	R	S	S	R	R	R	S	+	+	+	ø	ø	ø	ø	ø	ø	ø	ø	ø	ø	ø	ø	ø	ø	ø	ø	ø	+	+	+	ø	+	+
Kp54	U	R	R	R	R	R	R	R	R	R	R	R	R	R	R	+	+	+	ø	ø	ø	ø	ø	ø	ø	ø	ø	ø	ø	ø	+	ø	ø	ø	ø	+	+	ø	ø	+	+
Kp55	U	R	R	R	R	R	R	R	R	R	R	R	R	R	R	+	+	+	ø	ø	ø	ø	ø	ø	ø	ø	ø	ø	ø	ø	+	ø	ø	ø	ø	+	+	+	ø	+	+
Kp56	BC	R	R	R	R	R	R	R	R	R	R	R	R	R	R	+	+	+	ø	ø	ø	ø	ø	ø	ø	ø	ø	ø	ø	ø	+	ø	ø	ø	ø	+	+	+	ø	+	+
Kp57	BC	R	R	R	R	R	R	R	R	R	R	R	R	R	R	+	+	+	ø	ø	ø	ø	ø	ø	ø	ø	+	ø	ø	ø	+	ø	ø	ø	ø	+	+	+	ø	+	+
Kp58	BC	R	R	R	R	R	R	R	R	R	R	R	R	R	R	+	+	+	ø	ø	ø	ø	ø	ø	ø	ø	+	ø	ø	ø	+	ø	ø	ø	ø	+	+	+	ø	+	+
Kp59	BC	R	R	R	R	R	R	R	R	R	R	R	R	R	R	+	+	+	ø	ø	ø	ø	ø	ø	ø	ø	+	ø	ø	ø	+	ø	ø	ø	ø	+	+	+	ø	+	+
Kp60	BC	R	R	R	R	R	R	R	R	S	S	R	R	R	S	+	+	+	ø	ø	ø	ø	ø	ø	ø	ø	ø	ø	ø	ø	+	ø	ø	ø	ø	+	+	+	ø	+	+
Kp61	U	R	R	R	R	R	R	R	R	R	R	R	R	R	R	+	+	+	ø	ø	ø	ø	ø	ø	ø	ø	+	ø	ø	ø	+	ø	ø	ø	ø	+	ø	+	ø	+	+
Kp62	SPT	R	R	R	R	R	R	R	R	R	R	R	R	R	R	+	+	+	ø	ø	ø	ø	ø	ø	ø	ø	+	ø	ø	ø	+	ø	ø	ø	ø	+	+	+	ø	+	+

Abbreviations: BC, Blood culture; U, Urine; WC, Wound culture; SPT, Sputum; R, resistant; S, susceptible; +, gene detected; ø, gene not detected.
