# Supplementary material for: Clonal Relatedness and Transferable Resistance in Carbapenem-Resistant Klebsiella pneumoniae: An Integrated Molecular Epidemiological Study
Source: Microorganisms. 2026 Sep 29;14(10):2181. doi: 10.3390/microorganisms14102181 (PMC13707725; doi:10.3390/microorganisms14102181)
Supplement: Supplementary file 1 [file microorganisms-14-02181-s001.zip › Supplementary Table S4.pdf]

Supplementary Table S4. Minimum inhibitory concentrations (MICs) of ertapenem, imipenem, and meropenem in the 62 carbapenem-resistant *Klebsiella pneumoniae* clinical isolates.

İsolate No	Imipenem MIC (mg/L)	Imipenem	Ertapenem MIC (mg/L)	Ertapenem	Meropenem MIC (mg/L)	Meropenem
Kp1	>8	R	>4	R	>8	R
Kp2	>8	R	>4	R	>8	R
Kp3	>8	R	>4	R	>8	R
Kp4	>8	R	>4	R	>8	R
Kp5	>8	R	>4	R	>8	R
Kp6	>8	R	>4	R	>8	R
Kp7	>8	R	>4	R	>8	R
Kp8	>8	R	>4	R	>8	R
Kp9	>8	R	>4	R	>8	R
Kp10	>8	R	>4	R	>8	R
Kp11	>8	R	>4	R	>8	R
Kp12	>8	R	>4	R	>8	R
Kp13	>8	R	>4	R	>8	R
Kp14	>8	R	>4	R	>8	R
Kp15	>8	R	>4	R	>8	R
Kp16	>8	R	>4	R	>8	R
Kp17	>8	R	>4	R	>8	R
Kp18	>8	R	>4	R	>8	R
Kp19	>8	R	>4	R	>8	R
Kp20	>8	R	>4	R	>8	R
Kp21	>8	R	>4	R	>8	R
Kp22	>8	R	>4	R	>8	R
Kp23	>8	R	>4	R	>8	R
Kp24	>8	R	>4	R	>8	R
Kp25	8	R	>4	R	8	R
Kp26	>8	R	>4	R	>8	R
Kp27	>8	R	>4	R	>8	R
Kp28	8	R	>4	R	8	R
Kp29	>8	R	>4	R	>8	R
Kp30	>8	R	>4	R	>8	R
Kp31	>8	R	>4	R	>8	R
Kp32	>8	R	>4	R	>8	R
Kp33	>8	R	>4	R	>8	R
Kp34	>8	R	>4	R	>8	R
Kp35	>8	R	>4	R	>8	R
Kp36	>8	R	>4	R	>8	R
Kp37	>8	R	>4	R	>8	R
Kp38	>8	R	>4	R	>8	R
Kp39	>8	R	>4	R	>8	R
Kp40	>8	R	>4	R	>8	R
Kp41	>8	R	>4	R	>8	R
Kp42	>8	R	>4	R	>8	R
Kp43	>8	R	>4	R	>8	R
Kp44	>8	R	>4	R	>8	R
Kp45	>8	R	>4	R	>8	R
Kp46	>8	R	>4	R	>8	R
Kp47	>8	R	>4	R	>8	R
Kp48	>8	R	>4	R	>8	R
Kp49	>8	R	>4	R	>8	R

Kp50	>8	R	>4	R	>8	R
Kp51	>8	R	>4	R	>8	R
Kp52	>8	R	>4	R	>8	R
Kp53	>8	R	>4	R	>8	R
Kp54	>8	R	>4	R	>8	R
Kp55	>8	R	>4	R	>8	R
Kp56	>8	R	>4	R	>8	R
Kp57	>8	R	>4	R	>8	R
Kp58	>8	R	>4	R	>8	R
Kp59	>8	R	>4	R	>8	R
Kp60	>8	R	>4	R	>8	R
Kp61	>8	R	>4	R	>8	R
Kp62	>8	R	>4	R	>8	R

R: Resistant
